# Rapid compensatory evolution promotes the survival of conjugative plasmids

**DOI:** 10.1080/2159256X.2016.1179074

**Published:** 2016-05-04

**Authors:** Ellie Harrison, Calvin Dytham, James P. J. Hall, David Guymer, Andrew J. Spiers, Steve Paterson, Michael A. Brockhurst

**Affiliations:** a Department of Biology, University of York, York, UK; b SIMBIOS Center, Abertay University, Dundee, UK; c Institute of Integrative Biology, University of Liverpool, Liverpool, UK

**Keywords:** compensatory adaptation, experimental evolution, horizontal gene transfer, individual based model, transposition

## Abstract

Conjugative plasmids play a vital role in bacterial adaptation through horizontal gene transfer. Explaining how plasmids persist in host populations however is difficult, given the high costs often associated with plasmid carriage. Compensatory evolution to ameliorate this cost can rescue plasmids from extinction. In a recently published study we showed that compensatory evolution repeatedly targeted the same bacterial regulatory system, GacA/GacS, in populations of plasmid-carrying bacteria evolving across a range of selective environments. Mutations in these genes arose rapidly and completely eliminated the cost of plasmid carriage. Here we extend our analysis using an individual based model to explore the dynamics of compensatory evolution in this system. We show that mutations which ameliorate the cost of plasmid carriage can prevent both the loss of plasmids from the population and the fixation of accessory traits on the bacterial chromosome. We discuss how dependent the outcome of compensatory evolution is on the strength and availability of such mutations and the rate at which beneficial accessory traits integrate on the host chromosome.

## Introduction

Conjugative plasmids are vehicles for horizontal gene transfer, a key process in bacterial adaptation.^1^ The existence of conjugative plasmids however presents a paradox.^2^ Plasmid acquisition is often costly to the bacterial host as plasmids rely on host resources for their own replication, may disrupt host regulation, or have toxic effects on the cell, for instance through protein misfolding.^3^ Parasitic plasmids, which confer no benefit to their hosts, should therefore be lost from the population through purifying selection. By contrast, where plasmids carry useful accessory genes, positive selection may stabilize the plasmid in the population in the short term,^4^ but movement of accessory genes on to the bacterial chromosome breaks the linkage between plasmids and the trait under selection and thus the plasmid can be lost.^5^ Long-term selection experiments have demonstrated that evolution can help to solve this plasmid paradox. Conjugative plasmids can persist in experimental bacterial populations due to compensatory adaptation: mutations, whether occurring on the bacterial chromosome, the plasmid, or both,^6-16^ which reduce the cost of plasmid carriage and thus the strength of purifying selection can prolong plasmid survival. In a recent study we examined the dynamics of compensatory evolution across the continuum from parasitism to mutualism.^17^ The mega-plasmid pQBR103 carries a mercury resistance cassette, *mer*, itself carried on a transposon, which allows the bacteria to convert toxic mercuric ions, Hg(II), into less harmful elemental mercury. In the absence of Hg(II) however, plasmid carriage reduces the fitness of the host by ∼25%. Plasmid containing bacteria were experimentally evolved under 6 Hg(II) concentrations: from no Hg(II), where the plasmid is purely parasitic, to high levels of Hg(II) where the relationship is mutualistic.

**Figure 1.**
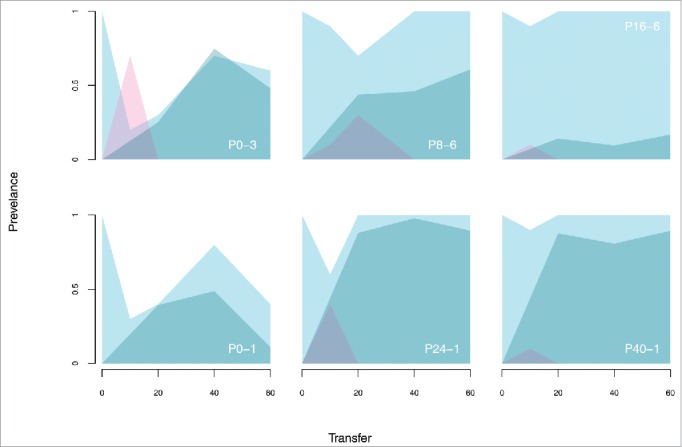
Dynamics of compensatory evolution in the empirical system. The frequency of the *gacA* or *gacS* mutation appearing in 6 of the 36 independently evolving populations was determined by allele frequency tracking. Shading indicates the prevalence of plasmid containing (blue) and chromosomal *mer* genotypes (red) and the frequency of amelioration mutations (dark blue). Population names are shown as ‘mercury treatment' – ‘replicate number'.

Counter to theoretical predictions, plasmids were maintained for over 450 generations across all environments, despite the potential for rapid plasmid loss in the absence of Hg(II) and transposition of the *mer* transposon onto the chromosome accompanied by plasmid loss under Hg(II) selection. Genome sequencing of evolved plasmid-containing clones revealed that mutations were exclusive to the bacterial chromosome. No mutations were found on evolved plasmids, with the exception of hypermutator strains that had acquired mutations in their mismatch repair pathway, and one clone with a transposon duplication. On the bacterial chromosome however we observed highly parallel evolution of the bacterial GacA/GacS 2-component response regulator. Excluding hypermutators, ∼80% of sequenced clones carried mutations in either *gacA* or *gacS*, with several clones sharing the exact nucleotide change and thus exhibiting parallelism at both the locus and nucleotide levels. In contrast, no *gacA* or *gacS* mutations were observed in plasmid-free control clones. Tracking the frequency of a subset of these *gacA* and *gacS* mutations through time revealed that they arose early and rapidly invaded the plasmid-containing fraction of the population (Fig. 1). Knock-out strains of these 2 genes confirmed that loss of GacA/GacS function completely ameliorated the cost of plasmid carriage.

Finally we examined the impact of plasmid acquisition and compensatory evolution on gene regulation. In the ancestral strain, plasmid carriage resulted in the up-regulation of ∼17% of the bacterial genome, including many genes associated with protein production processes, such as ribosome biogenesis and protein folding. These data indicate that plasmid carriage places a strong translational burden on the ancestral host, driving greater investment in the machinery of protein production. Following compensatory evolution, however, bacterial gene expression resembled the plasmid-free ancestor, suggesting that *gacA/gacS* mutations had relieved this translational burden.

The GacA/GacS system positively regulates the translation of a suite of bacterial secondary metabolites and secretions.^18^ The exact mechanism linking these mutations with the amelioration of the cost of pQBR103 carriage remains unclear. Loss of GacA/GacS function reduces the overall translation load of the cell by leaving in place repression of a large number of bacterial genes. In addition gene expression analysis revealed that ∼17% of plasmid genes were also down-regulated following compensatory evolution. Thus amelioration may be due to a general reduction in translation load on the cell and/or from specific down-regulation of plasmid genes, for instance those encoding cytotoxic misfolded proteins.^3^

Our study demonstrates the capacity for compensatory evolution to rescue a costly plasmid from extinction across a broad range of environments. Importantly, plasmids were maintained despite the early appearance of genotypes predicted to outcompete plasmid-carriers, specifically, plasmid free cells rapidly arose in the absence of mercury, and genotypes with chromosomal *mer* that had lost the plasmid arose in all Hg(II) environments. These genotypes were detectable at the first sampling point in 16 out of 36 populations despite relatively low resolution of sampling, unlikely to detect genotypes at frequencies below 10%. Thus by reducing purifying selection acting on the plasmid backbone, compensatory mutations were able to hinder the spread of these alternative genotypes and preserve the mobility of mercury resistance genes which may otherwise become lost or rendered non-conjugative on the bacterial chromosome.

### How much can we generalize these findings across bacteria-plasmid systems?

**Figure 2.**
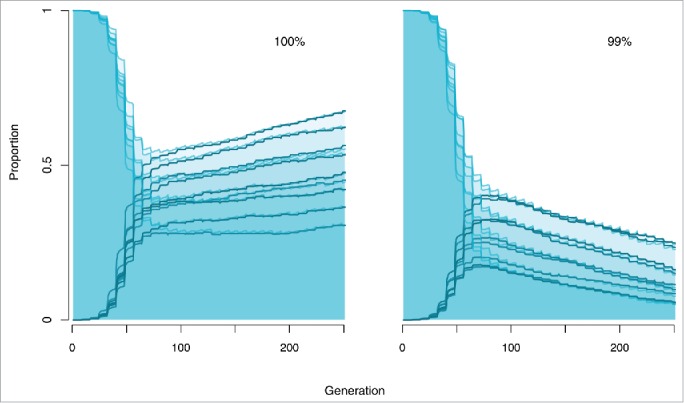
Effect of the degree of amelioration on the dynamics of compensatory evolution in the absence of positive selection. Each plot shows 9 individual iterations of the IBM with the proportion of plasmid carrying genotypes shown in light blue with shading, and the frequency of the amelioration allele shown in dark blue. The degree to which the amelioration mutation reduced the cost of the plasmid was varied from 100% (left) to 99% (right). Mutations which completely ameliorate the cost of the plasmid were able to rescue plasmids from extinction, while mutations which merely reduce it slowed but did not prevent plasmid loss.

In the study described, compensatory evolution was characterized by highly repeatable, rapid evolution of a global bacterial regulator, GacA/GacS. Knockout mutants demonstrate that loss of function in either gene is able to completely ameliorate the cost of the plasmid allowing strong selection on single mutations. In addition, evidence from *Pseudomonas* sp. PCL1171 suggests that *gacA/gacS* loci may in fact have elevated mutation rates in pseudomonads, estimated to be ∼100 times that of the genome-wide mutation rate.^19^ The large fitness effects and high mutation rate at these loci helps to explain the high-level of parallelism observed across replicates and environments in our experiment. To explore how dependent the outcome of our evolution experiment is on the nature of these compensatory mutations we produced an individual based model (IBM) capable of capturing the dynamics of amelioration, plasmid prevalence and transposon capture by the chromosome. The IBM is adapted from Harrison et al 2015^20^ and parameterized, where possible, using estimates taken from the empirical system. We first investigated the importance of the degree of amelioration. In the absence of Hg(II) selection, complete (100%) amelioration of the cost of carriage was required for plasmid survival in the population (Fig. 2). Where mutations reduced the cost of plasmid carriage by only 99%, plasmid loss was slowed but not halted suggesting that the balance between the underlying processes of plasmid loss via segregation and plasmid gain by conjugation is such that very slight purifying selection acting on plasmid carriage leads to a net decline in plasmid prevalence.

**Figure 3.**
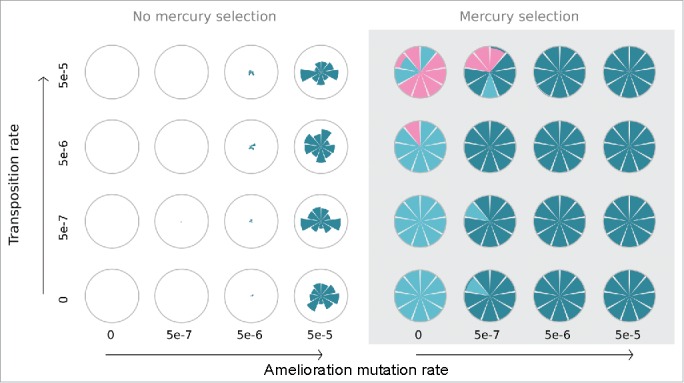
The impact of amelioration and transposition rate on the outcome of the IBM. Models were run across a range of amelioration mutation (x axis) and transposition (y axis) rates in the presence (right panel) and absence (left panel) of mercury selection. Nine individual iterations of the IBM were run for each combination of parameters. The composition of each population after 251 generations of the IBM is represented by each sector of a circle. Shading shows the proportion of plasmid-carrying genotypes (blue), compensatory mutations (dark blue) and plasmid-free, transposon-carrying (red) genotypes.

We next investigated the impact of mutation rate on the outcome of bacteria-plasmid dynamics. As observed in our empirical system, complete compensatory mutations were able to rescue plasmids from extinction even in the absence of positive selection for the plasmid (Fig. 3). However under these conditions (i.e. in the absence of Hg(II) selection) this outcome was strongly dependent on a high mutation rate. At mutation rates equivalent to estimates for the *gacA/gacS* loci (∼5×10^−5^) compensatory mutations arose in time to stabilize plasmid prevalence before the plasmid could be lost from the population. At lower mutation rates however the plasmid was generally lost before compensatory mutations were able to arise and invade (Supp figure).

In the presence of Hg(II) toxicity, positive selection for the *mer* operon maintained plasmids in the population in the short-term but, in the absence of compensatory mutations, plasmid carriers were ultimately out competed by plasmid free, *mer* carrying individuals, which do not pay the cost of plasmid carriage (Fig. 3). Our model suggests that by ameliorating the cost of plasmid carriage and thus negating the benefit of chromosomal *mer* acquisition, compensatory evolution can block this process. Where amelioration was allowed, compensatory mutations became fixed in almost all iterations of the IBM across a broad range of parameters before chromosomal *mer* genotypes became established. Only under conditions where the rate of transposition was relatively high (5×10^−5^)^21^ and amelioration mutation rate low (5×10^−7^) were chromosomal *mer* genotypes able to become established in the population (Fig. 3). This disparity can be understood as the advantage of chromosomal *mer* capture is only gained once the plasmid is lost, making it a 2-step process and thus less likely than transposition events alone.

### Toward resolving the plasmid paradox

The persistence of plasmids in bacterial populations has been challenging to explain because, while mechanisms to increase plasmid stability are widespread, they are far from universal^16^ and rarely sufficient to prevent plasmid loss given the costs of plasmid carriage. Recent experimental data and our model show that rapid compensatory evolution is key to understanding this puzzle, allowing plasmids to be maintained in the long-term by alleviating purifying selection acting on the plasmid. As demonstrated by our simulation model the long-term fate of plasmids in bacterial populations ultimately comes down to a race between competing processes; plasmid loss, the capture of accessory genes by the bacterial chromosome and the appearance of compensatory mutations. By slowing the rate of plasmid loss, stability mechanisms (such as toxin/antitoxin systems^22^), high conjugation rates (for instance promoted by structured environments^23^) or low initial cost of plasmid carriage will increase the probability of compensatory evolution occurring in time to rescue plasmids in the population.

Our model also highlights the role for positive selection acting on plasmid-borne traits in expanding the conditions under which compensatory evolution is likely to occur. As plasmid loss is contingent first on the movement of accessory genes onto the bacterial chromosome the resulting delay allows even rare amelioration mutations to appear and spread within the plasmid population. Indeed even very transient positive selection, by increasing plasmid population size, can facilitate the establishment of compensatory mutations and stabilization of initially costly plasmids in bacterial populations.^16^

In the absence of positive selection however, we find that the conditions for plasmid maintenance are surprisingly stringent; amelioration mutations must have both large fitness effects and occur at a high rate in order to stabilize plasmids in bacterial populations. That such large-effect, high-frequency mutations appeared in our experimental system may therefore merely be good fortune, but it is possible that such mutations are more readily available than might be expected. At present we have a limited understanding of what causes the costs of plasmid carriage and thus it is hard to predict which mutations might be available to ameliorate their cost. Recent evidence however suggests that, in addition to more general costs of plasmid maintenance (i.e., in competing with the chromosome for cellular resources), plasmid costs are frequently associated with specific, acute maladaptations, for instance strong epistatic interactions between bacterial and plasmid bound genes allowing single mutations to have large fitness effects.^24^ It is notable that a number of experimental evolution studies report that compensatory adaptation is often associated with the bacterial chromosome.^6,7,16,17^ As illustrated by the *gacA/gacS* mutations observed in our empirical study, bacterial genomes can be highly flexible. The GacA/GacS system has been suggested to act as a contingency loci for environmental adaptation, where high mutation rates allow for phenotypic switching by mutation and reversion.^25^ Such evolutionary control of regulatory systems may be equally important for dealing with perturbations to the intracellular environment caused by HGT as for adapting to changes to the external environment.

### Future directions

To understand the dynamics of plasmids in bacterial populations we must therefore view bacteria-plasmid associations through both an ecological and evolutionary lens. While the initial invasion of plasmids into bacterial populations may be dependent on the specifics of the bacteria x plasmid x environment interaction, compensatory evolution allows plasmids to be stably maintained in the absence of positive selection, preserving a reservoir of mobile accessory genes and allowing potential for adaptation by horizontal gene transfer. The implications of these evolutionary changes for bacterial and plasmid populations are a rich subject for future investigations. As yet we know very little about the types of mechanisms that underlie the costs of plasmid carriage and consequently the avenues to their amelioration. Do compensatory mutations lead to tradeoffs in bacterial fitness in other environments, as might be expected in the case of the GacA/GacS loci? Are these adaptations specific to the coevolving bacteria-plasmid association? Or do compensatory mutations in the host ameliorate the cost of other plasmids in the community, producing more generalist hosts? Understanding what consequences compensatory evolution might have for both bacteria and plasmids will therefore be key in understanding the persistence of plasmids in natural microbial communities.

## Supplementary Material

KMGE_S_1179074.zip
