# Supplementary material for: Rapid compensatory evolution promotes the survival of conjugative plasmids
Source: Mob Genet Elements. 2016 May 4;6(3):e1179074. doi: 10.1080/2159256X.2016.1179074 (PMC4964889; doi:10.1080/2159256X.2016.1179074)
Supplement: KMGE_S_1179074.zip [file kmge-06-03-1179074-s001.zip › KMGE_S_1179074.docx]

Supplementary figure. Population dynamics for 9 iterations of the IBM exploring the impact of varying amelioration and transposition rates on plasmid persistence in the absence and presence of mercury selection. Plots show the prevalence of plasmid carriers (blue), amelioration mutations (dark blue) and plasmid-free, transposon-carrying (red) genotypes through time. For each plot parameters for amelioration mutation rate, transposition rate and mercury selection was varied as specified by the outer axes.
